# Meta-Analysis of Prevalence of Depression in Dental Students during COVID-19 Pandemic

**DOI:** 10.3390/medicina57111278

**Published:** 2021-11-21

**Authors:** Javier Santabárbara, Naiara Ozamiz-Etxebarria, Nahia Idoiaga, Beatriz Olaya, Juan Bueno-Novitol

**Affiliations:** 1 Department of Microbiology, Pediatrics, Radiology and Public Health, University of Zaragoza, C/Domingo Miral s/n, 50009 Zaragoza, Spain; jsantabarbara@unizar.es; 2 Centro de Investigación Biomédica en Red de Salud Mental (CIBERSAM), Ministry of Science and Innovation, 28029 Madrid, Spain; beatriz.olaya@sjd.org; 3 Aragonese Institute of Health Sciences (IIS Aragón), 50009 Zaragoza, Spain; 4 Department of Developmental and Educational Psychology, University of the Basque Country UPV/EHU, 48940 Leioa, Spain; naiara.ozamiz@ehu.eus; 5 Research, Innovation and Teaching Unit, Parc Sanitari Sant Joan de Déu, Universitat de Barcelona, 08830 Barcelona, Spain; 6 Psychiatry Service, Hospital Universitario Miguel Servet, 50009 Zaragoza, Spain; elecrijuan@hotmail.com

**Keywords:** depression, dental students, gender, countries, meta-analysis, COVID-19

## Abstract

*Background and Objectives*: The COVID-19 pandemic has a negative impact on the mental health of the population in general, and in college students in particular. Dental students have seen their teaching altered and their clinical practice reduced. This study was aimed at conducting a systematic review and meta-analysis of studies reporting levels of depression among dental students during the COVID-19 and estimating the pooled prevalence of depression. *Materials and Methods*: Medline via PubMed and other databases were searched for studies on the prevalence of depression in dental undergraduates, published from 1 December 2019 to 1 September 2021. The pooled proportions of depression were calculated with random effects models. *Results*: We identified 13 studies from 9 countries. The pooled prevalence of depression in dental students was 37% (95% CI: 26–49%) with no variation due to gender, response rate or methodological quality. We only found a significantly higher prevalence of depression in studies from Asia compared to Europe and America. *Conclusions*: Our results suggest that dental students are suffering from higher levels of depression compared with the general population or other college students during the COVID-19 pandemic, with differences across regions. Measures to improve mental health and wellbeing of dental students during the pandemic are needed.

## 1. Introduction

Since March 2021, when the World Health Organization (WHO) declared the COVID-19 pandemic [1,2], there have been profound economic, social, psychological and educational changes worldwide [3]. However, the pandemic has not equally impacted different segments of the population [4]. Specifically, several target groups have been studied for the negative psychological impact of the COVID-19 [5]. Among them, university students have concerned the international scientific community [6]. One of the first measures to contain the pandemic, and to stop the spread of the virus, was to close universities, with considerable associated psychological symptoms in this population [7]. University education had to be transformed overnight into a virtual mode [8], and students had to quickly adapt to this change. Students had to deal with delays in academic activities [9], the transition to online education, the administration of homework, projects and other ongoing assessments. Students began to fear that the pandemic could have a serious impact on their careers [10,11] and they were also very concerned about their health, safety and the well-being of their families [12]. Social distancing, lockdowns and other restrictions and norms to stop the spread of the virus are factors related to an increased rates of depression in the general population [13] and particularly in university students [14,15]. This increased level of depression seems to be related to the fear of getting infected and adaptation difficulties to personal, academic and professional restrictions [16,17].

Previous studies have shown that dental students have important levels of depression before the pandemic [18,19,20]. This previous psychological distress could be caused by the high emotional burden of dental studies due to long study hours, high workload [21], clinical demands, examinations and qualifications [22,23]. Moreover, the levels of depression seem to increase as the years of medical school progress, showing higher symptomatology than medical students [24,25]. In addition, dental students in their clinical practice are especially vulnerable to the risk of COVID-19 transmission [26], as they have to work closely with patients. This proximity may put them at a higher risk of viral exposure and being infected with COVID-19 [27]. In fact, droplets and aerosols produced during most dental procedures are potential methods for COVID-19 transmission [28].

Research has suggested several sociodemographic variables that might be associated with higher rates of depression during the COVID-19 pandemic. In general, higher rates of depression have been observed among females [29,30,31], although there are studies suggesting that there are no differences between men and women [32,33]. In addition, significant differences have also been found in the prevalence of depression among university students depending on the country [6,34].

Several reports, opinion articles and studies have recently been published about the psychological impact of the COVID-19 pandemic on dental students. However, to the best of the authors’ knowledge, there are no studies that synthesize the current scientific evidence on this topic.

Thus, the main objective of this study is to conduct a systematic review and meta-analysis of studies that investigate the prevalence of depression in dental students during the COVID-19 pandemic. Moreover, it will be analyzed whether gender, age or the country were the study was carried out create significant differences in the prevalence of depression among dental students.

## 2. Materials and Methods

This study follows the methodology of a previous work [35], and was conducted in accordance with the PRISMA guidelines for reporting systematic reviews and meta-analysis [36] (Supplementary Table S1).

### 2.1. Search Strategy

Two researchers (JS and JBN) searched for all cross-sectional studies reporting the prevalence of depression published from 1 December 2019 to 1 September 2021, using MEDLINE via PubMed, Embase and Scielo databases. The search strategy used in PubMed is detailed in Table 1.

No language restriction was made. References from selected articles were inspected to detect additional potential studies. Then, we performed a manual search of the “grey literature” (e.g., medRxiv or Google Scholar) to detect other potentially eligible papers. In addition, the reference lists of selected publications were also screened for potentially eligible studies. Authors of studies were contacted directly when insufficient data were available in articles meeting the inclusion criteria or the full text was not available.

### 2.2. Selection Criteria

Studies were included if: (1) reported cross-sectional data on the prevalence of depression, or sufficient information to compute this, conducted during the COVID-19 outbreak; (2) focused on dental students; and (3) included a validated instrument to assess or diagnose depression.

We excluded studies focusing only on community-based samples of general population or specific samples that were not dental students (e.g., medical students, medical professionals, patients), as well as review articles.

A pre-designed data extraction form was used to extract the following information: country, sample size, prevalent rates of depression, proportion of women, average age, instruments used to assess depression, response rate and sampling methods.

### 2.3. Methodological Quality Assessment

Articles selected for retrieval were assessed by one reviewer (JBN) for methodological validity before they were included in the review using the Joanna Briggs Institute (JBI) standardized critical appraisal instrument for prevalence studies [37]. Quality was evaluated according to nine criteria, each yielding a score of zero or one. One score was obtained for each criterion if the study was affirmative in the next questions: 1: Was the sample frame appropriate to address the target population? 2: Were study participants recruited in an appropriate way? 3: Was the sample size adequate? 4: Were the study subjects and setting described in detail? 5: Was data analysis conducted with sufficient coverage of the identified sample? 6: Were valid methods used for the identification of the condition? 7: Was the condition measured in a standard, reliable way for all participants? 8: Was there appropriate statistical analysis? 9: Was the response rate adequate, and if not, was the low response rate managed appropriately?

### 2.4. Data Extraction and Statistical Analysis

Freeman and Tukey’s double arcsine transformation of prevalence to stabilize the variance was applied [38]. A generic inverse variance method with a random effect model was used [39]. The Hedges *Q* statistic was reported to check heterogeneity across studies, with statistical significance set at *p* < 0.10. The *I*^2^ statistic and 95% confidence interval was also used to quantify heterogeneity [40]. Values between 25 and 50% are considered as low, 50 and 75% as moderate, and 75% or more as high [41]. Heterogeneity of effects between studies occurs when differences in results for the same exposure–disease association cannot be fully explained by sampling variation. Sources of heterogeneity can include differences in study design or in demographic characteristics. We performed meta-regression and subgroup analyses [42] to explore the sources of heterogeneity expected in meta-analyses of observational studies [43]. We conducted a sensitivity analysis to determine the influence of each individual study on the overall result by omitting studies one by one. Publication bias was determined through visual inspection of a funnel plot and also Egger’s test [44] (*p* values < 0.05 indicate publication bias) since funnel plots were found to be an inaccurate method for assessing publication bias in meta-analyses of proportion studies [45].

Statistical analyses were conducted by one researcher (JS) and run with STATA statistical software (version 10.0; College Station, TX, USA) and R [46].

## 3. Results

**Figure 1:**
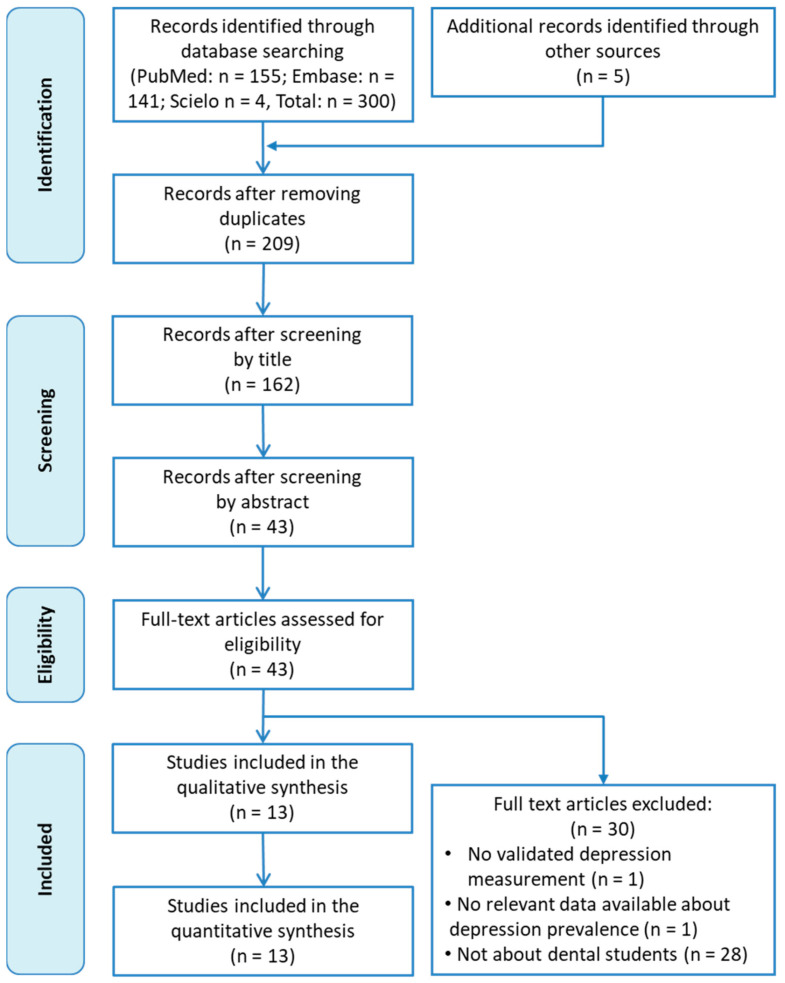
Flowchart of the study selection.

Figure 1 shows the flowchart of the literature search strategy and study selection process. In total, 300 records were initially identified from Medline via PubMed, Embase and Scielo, and five extra records were then added after a manual search in a preprints database (MedRxiv) and Google Scholar. A total of 96 duplicate articles were deleted and 166 were excluded after a first screening of the titles and abstracts. After reading the remaining 43 articles in full, we finally included 13 in our meta-analysis [47,48,49,50,51,52,53,54,55,56,57,58,59]. Exclusion reasons are detailed in Figure 1.

Table 2 shows a description of the studies included. Most of the studies were conducted in Asia (*n* = 10), but we also found studies from Europe (*n* = 1), North (*n* = 1) and South America (*n* = 1), with sample sizes ranging from 97 to 699 participants. Most of the studies involved young students, and gave data referring to the academic year, while only six articles reported the mean age of participants, which ranged from 21.31 to 25.10 years. All studies included both men and women, with a clear predominance of women in all studies. All studies were conducted using online questionnaires and, of those reporting the sampling methodology, all except two used non-random methods. The response rate was reported by eight studies and ranged from 20% to 95.10%. All studies measured depression using standardized scales, most commonly the Depression, Anxiety and Stress Scale (DASS, *n* = 10 studies) and the Patient Health (PHQ, *n* = 2 studies), with one study using the Hospital Anxiety and Depression Scale (HADS).

Risk of bias scores ranged from 5 to 9 out of a possible total of 9, with a mean score of 6.7 (SD = 1.2) (Supplementary Table S2). The most common limitations were: (a) recruitment of participants not appropriate (11 studies), (b) sample size too small to ensure good precision of the final estimate (9 studies), and (c) response rate not reported, or large number of non-responders (6 studies).

**Figure 2:**
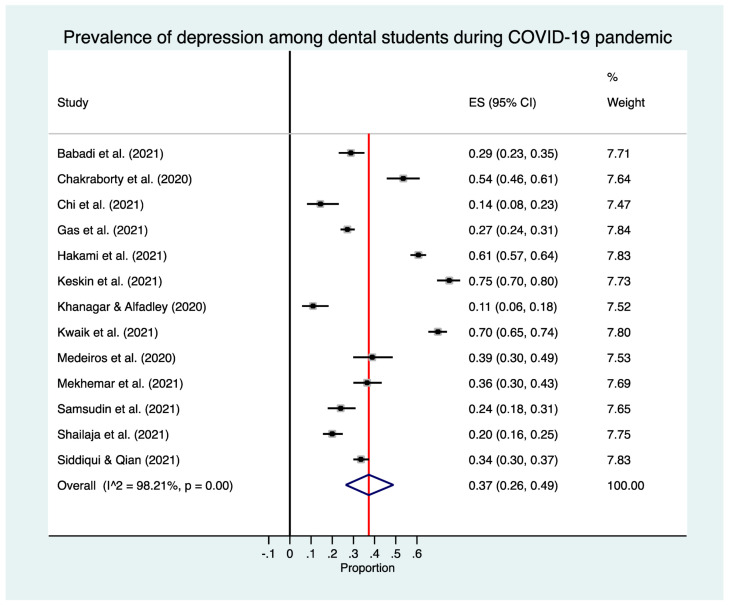
Forest plot for the prevalence of depression in dental students.

The estimated overall prevalence of depression was 37% in dental students (95% CI: 26–49%), with significant heterogeneity between studies (Q test: *p* < 0.001; *I*^2^ = 98.3%) (Figure 2).

Our meta-regression analysis showed that the prevalence rate of depression was independent of the percentage of women (*p* = 0.815), mean age at baseline (*p* = 0.407), response rate (*p* = 0.727) or methodological quality (*p* = 0.847). According to subgroup analysis, the only relevant finding was a higher prevalence of depression for studies conducted in Asia (40% (95% CI: 27–53%)) compared to those from Europe or America (29% (95% CI: 16–45%)). We also observed higher prevalence of depression for studies using the PHQ-9 (38% (95% CI: 32–44%)) compared to those using the DASS (−21 or −42) (38% (95% CI: 25–52%)) or HADS (39% (95% CI: 30–49%)); and those using random or cluster sampling methods (44% (95% CI: 41–46%)) compared to those using convenience sampling method (36% (95% CI: 24–50%)).

Excluding each study one-by-one from the analysis did not substantially change the pooled prevalence of depression, which varied between 35% (95% CI: 24–46%), with Keskin et al. [52] excluded, and 40% (95% CI: 29–52%), with Khanagar & Alfadley [53] excluded. This indicates that no single study had a disproportional impact on the overall prevalence.

**Figure 3:**
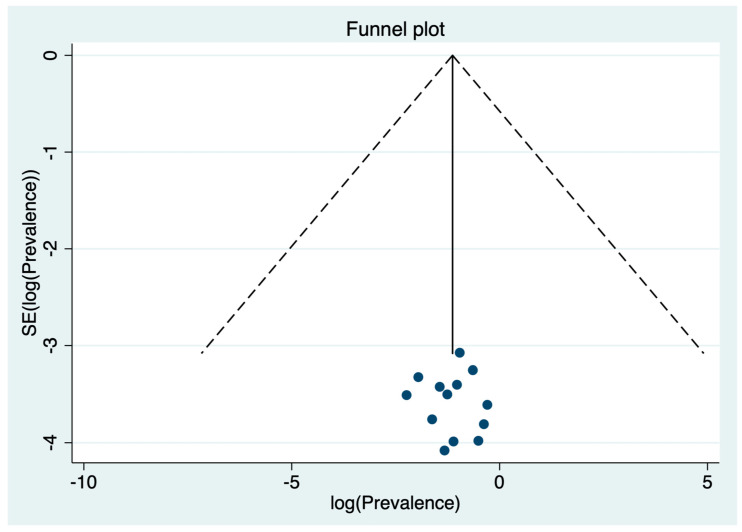
Funnel plot for the prevalence of depression in dental students.

Visual inspection of the funnel plot (Figure 3) suggested no publication bias for the estimate of prevalence of depression in dental students, confirmed by a non-significant Egger test (*p* = 0.679).

## 4. Discussion

### 4.1. Summary of Main Findings

Depression has become one of the most important psychological consequences of the COVID-19 pandemic among university students in general, and dental students in particular. To the best of the authors’ knowledge, this is the first meta-analysis reporting pooled depression in dental students during the COVID-19 pandemic. Based on 13 studies, we estimated an overall prevalence of depression of 37%, with higher prevalence of depression found in Asian samples.

Among the studies analyzed, the one that found a lower prevalence of depression among dental students was the one conducted by Khanagar & Alfadley. [53] in 2020 in Saudi Arabia with a prevalence of 20.9%. However, Hakami et al. [51] found a prevalence of 60.7% in the same country in 2021. On the other hand, the highest rate of depression among dental students, 75.3%, was found by Keskin et al. [52] in Turkey in 2021.

Previous meta-analyses on the levels of depression in the general population during the pandemic have reported prevalence rates between 22.8% [60] and 33.7% [61]. As for university students, a great variability has been reported in terms of depressive symptomatology. Previous reviews found that the prevalence rate of depression during the pandemic ranged between 12.2% [62] and 31.2% [6]. However, more recent reviews conducted in 2021 suggest that these rates could be up to 32% [63] and 37% [64].

Our results are consistent with these previous works suggesting that college students, and dental students in particular, are suffering from higher levels of depression than the general population [65]. Moreover, this review also points out that the prevalence of depression among dental students seems to be higher than that observed for university students in general, something that had also been found in studies prior to the pandemic [25,66].

When analyzing differences according to several characteristics, we did not find significant differences by gender or age. These results are not in line with other studies conducted in the general population that found higher rates of depression in women compared to men during the COVID-19 pandemic [29]. This lack of differences may be due to the characteristics of university students, usually young and without family responsibilities. These factors could in turn be risk factors for depression in in women from other types of populations [67,68]. As for the age, one study found that undergraduated students showed higher rates of depression compared with graduated students [69], although they found no significant difference according to the undergraduate year. Our results suggest that in dental students the prevalence of depression does not seem to depend on the age of participants.

According to our findings, the most significant difference of depression was found between geographical regions. In fact, there seems to be a higher prevalence of depression in studies conducted in Asia (42%) compared to those located in Europe or America (27%). This is in line with another meta-analysis conducted in the general population, where the highest prevalence of depression was found in Asia [61] and another that found the highest incidence of depression in the Chinese and Italian general population [70]. In contrast, a previous meta-analysis conducted with the general population and university students found that Asia was the region with the lowest prevalence of depression [6].

The fact that we found a greater proportion of depression among Asian dental students could be explained by differences in the education system across regions. In an international study conducted by Perry et al. in 2017 [71] on simulation and curriculum design in dental studies, it was found that Asian dental colleges were more likely to use alternative methods of learning, such as haptic simulations, the Phantom Laboratory or any simulated teaching aids compared with Europe or America. Since many of these simulated motivating practices had to stop due to the lockdown and social distancing measures during the COVID-19 pandemic, dental studies had to become more traditional for Asian students. This might have increased their work overload and traditional examinations, which are a known major source of depression for dental students [24].

The COVID-19 pandemic seems to be long-lasting, with a significant psychological burden for people. Thus, it is important to stablish preventive and intervention programs to reduce this toll, especially among more vulnerable subgroups, such as dental students. It is also important to continue collecting data about the impact of the COVID-19 on dental students, especially in countries where these data are still not available.

### 4.2. Strengths and Limitations

The main strength of this study is that it is the first meta-analysis performed about the prevalence of depression in dental students during COVID-19 pandemic. Moreover, this study includes of a large body of literature and it uses of a rigorous approach to identify publication bias (i.e., Egger’s test), concluding that there is no bias in the estimation of the pooled prevalence of depression for dental students.

However, this study also has some limitations that should be taken into account. Firstly, the majority of the studies included in the systematic review were based on non-probabilistic samples, but no longitudinal studies were found. Therefore, we do not have scientific evidence to conclude whether the levels of depression have been sustained, reduced or increased over pandemic months [31]. Secondly, the studies included in this study used variety of self-reported standardized questionnaires, being this as a common practice in epidemiological studies [18,72]. However, our results have found that the use of some of these tests was associated with significantly higher prevalence of depression than others. Therefore, ideally, studies should use the same measure of depression and if possible, include a diagnosis based on clinical interviews. Finally, while we were able to include studies from different continents, there were only two studies from the Americas and one from Europe. It would therefore be advisable to carry out more studies in these countries, since there seems to be significant differences depending on the region of study.

## 5. Conclusions

This meta-analysis concludes that the prevalence of depression among dental students during the COVID-19 pandemic is high, with Asian students showing higher rates than non-Asian students. Taking into account that university students in general and dental students in particular are suffering from higher depressive symptoms, the lack of interventions and policies to improve mental health at universities may bring short and long-term psychological consequences in their personal, academic and professional life [73]. Therefore, universities should also work to adapt their teaching methods to this new situation. For dental studies, for example, it might be convenient to strengthen alternative methods of learning, such as haptic simulations or other simulated teaching, along with blended learning and virtual curriculum [74].

## Figures and Tables

**Table 1:** Search strategy in Pubmed.

(COVID [tiab] OR COVID-19[tiab] OR coronavirus[tiab] OR SARS-CoV-2[tiab] OR “Coronavirus”[Mesh] OR “severe acute respiratory syndrome coronavirus 2”[Supplementary Concept] OR “COVID-19”[Supplementary Concept] OR “Coronavirus Infections/epidemiology”[Mesh] OR “Coronavirus Infections/prevention and control”[Mesh] OR “Coronavirus Infections/psychology”[Mesh] OR “Coronavirus Infections/statistics and numerical data”[Mesh]) AND (“Depression”[Mesh] OR “Depressive Disorder”[Mesh] OR “depression”[tiab] OR “depressive”[tiab] OR “Depression/statistics and numerical data”[Mesh]) AND (“Students, Dental”[Mesh] OR “dental students”[tiab] OR “dentistry students”[tiab] OR “dental undergraduates”[tiab] OR “dentistry undergraduates”[tiab] OR “university students” [tiab])

**Table 2:** Description of studies included in meta-analysis.

Author (Publication Year)	Country	Mean Age (SD)	% Females (*n*)	Sample Size (*n*)	Response Rate (%)	Sampling Method	Depression Assessment	Diagnostic Criteria	Prevalence		Quality Assessment *
									%	*n*	
Babadi et al. (2021) [47]	Iran	22.9 (3.3)	53.28% (122)	229	54.5%	Convenience sampling	DASS-21	≥10	28.8%	66	7
Chakraborty et al. (2020) [48]	India	24 (3)	81.55% (137)	168	NR	Convenience sampling	PHQ-9	≥10	53.5%	90	6
Chi el al. (2021) [49]	USA	NR	52.58% (51)	97	35.5%	Convenience sampling	PHQ-9	≥10	14.4%	14	6
Gaș et al. (2021) [50]	Turkey	21.31 (1.9)	64.66% (452)	699	95.1%	Random sampling	DASS-21	≥10	27.2%	190	9
Hakami et al. (2021) [51]	Saudi Arabia	21.76 (1.9)	54.82% (381)	695	NR	Cluster sampling	DASS-21	≥10	60.7%	422	8
Keskin et al. (2021) [52]	Turkey	NR	60.23% (156)	259	NR	Convenience sampling	DASS-42	≥10	75.3%	195	5
Khanagar & Alfadley (2020) [53]	Saudi Arabia	25.1 (NR)	64.55% (71)	110	68.7%	Convenience sampling	DASS-21	≥10	10.9%	12	7
Kwaik et al. (2021) [54]	Palestine	NR	81.19% (354)	436	55.18%	NR	DASS-21	≥10	69.9%	305	8
Medeiros et al. (2020) [55]	Brazil	21.46 (2.37)	76.99% (87)	113	51.36%	NR	HADS	≥8	38.9%	44	6
Mekhemar et al. (2021) [56]	Germany	NR	73.46% (155)	211	NR	Convenience sampling	DASS-21	≥6	26.5%	77	5
Samsudin et al. (2021) [57]	Malaysia	NR	79.43% (139)	175	94.6%	Convenience sampling	DASS-21	≥10	24%	42	6
Shailaja et al. (2021) [58]	India	22.63 (2.88)	82.00% (246)	300	NR	NR	DASS-21	≥10	20%	60	7
Siddiqui & Qian (2021) [59]	Malaysia	22.45 (NR)	79.24% (519)	655	20%	Convenience sampling	DASS-21	≥10	33.6%	220	7

Note. * Quality score based on the Joanna Briggs Institute (JBI) standardized critical appraisal instrument for prevalence studies [Moola et al., 2017]. DASS = Anxiety, Anxiety and Stress scales; HADS = Hospital Anxiety and Depression Scale; NR = not reported; PHQ-9 = Patient Health Questionnaire; USA = United States of America.

## Data Availability

Not applicable.

## Supplementary Materials

The following are available online at https://www.mdpi.com/1648-9144/57/11/1278/s1, Table S1: PRISMA Checklist, Table S2: Quality assessment.
